# Should the Garden Cities Be Extended?

**Published:** 1919-08-16

**Authors:** 


### SOME PENALTIES OF TOWN LIFE.

### Should the Garden Cities be Extended?

The conditions of life amongst crowded communities may well be the subject of serious thought to those of us who interest ourselves in the welfare of the individual, and, therefore, of the nation.

There are still great numbers of rooms into which the sunlight never penetrates, and whose vitiated atmospheres form a potent *nidus* for the growth of the pathological micro-organism. These rooms are to be found in every class of house, though most so in the "slums" . . . which, unfortunately, are as much present in the West End of London as in the East End, in quality, though not in quantity. The "area," that proverbial hotel of the guardians of the law, so often leads into a basement consisting of one or more rooms whose outlook boasts no more than a whitewashed wall . . . with a coal-cellar and w.c. adjacent. Even when the window is opened to admit of fresh air everything in that room is soon coated with dust, and the dust of our cities is a very dangerous enemy; less so since motor traction appeared, but even yet enough to infect with most serious disease—note the great numbers of cases of septic and other varieties of tonsilitis and rhinitis in hot, dry weather.

### Evil of Overcrowding.

With the poorer houses the conditions for evil are much more marked, for here we have another added factor—viz., overcrowding. Imagine a worker of either sex, arriving home tired out, and, therefore, with lowered resistance to disease . . . to spend the hours of recreation and repose in an atmosphere that could never be called "virgin," but compounded of emanations of every kind and description. Small wonder then that he soon lacks vital energy—for oxygen is our greatest medical friend. True it is that we have our parks and open spaces in all large cities, but the possession of a private garden, however small, is of greater value to a tired mortal.

Spend a week in the heart of London, especially near a goods station or similar place, where the heavy-wheeled traffic beneath your window barely ceases day or night. Take a walk from the Bank to Shepherd's Bush . . . it is not distance that has tired you, for you will walk often much farther to reach a mountain-top without half the fatigue, it is, indeed, the constant jarring and discordant sounds which are irritating the cochlear nerves almost past endurance. A discord is fatiguing, a concord is refreshing. Thanks largely to the war, the strain and stress of modern town life has become more and more noticeable, and neurasthenia more and more prevalent. To watch a crowd, of almost every description, fight for an already crowded omnibus or train, to note the savage fury of the would-be passenger as he, or she (and she is invariably the worse offender), pushes to force a way, is to realise the mental state induced by modern conditions.

### Disease and Surroundings.

Another matter of which we hear a great deal to-day is the question of diseased tonsils and adenoids, and treatment, both prophylactic and curative; but the predisposing elements to this condition are partly to be found in the surroundings. All doctors dealing with these cases must agree that, relatively, many more town children come to them for treatment of this disease than children living in the country districts. This is a most important point, for diseased tonsils and adenoids form the breeding grounds of many of our most dangerous pathological micro-organisms.

It is quite certain that there is no more efficient distributor of bacteria than the crowded omnibus or underground train, for during the busy hours of ingress to and exit from the different places of business it is possible to realise fully a condition of early asphyxiation when standing in a closed compartment packed full. To the many thousands already predisposed to the invasion of tubercle the increased risks are only too apparent.

He only who has tasted the crisp fresh vegetable, or sucked the juicy fruit fresh plucked from a country garden, or replenished his gastric vacuity with a slice of fresh mountain mutton, can realise to the full the meaning of artefact and adulteration as applied to the food supplied in our great cities.

### The Loss of Young Lives.

It is truly tragical to learn how many are the valuable young lives lost to the community through the conditions which we have attempted to describe. This, too, at a time when it is absolutely vital to the country that we should have robust health and strength to retain for Great Britain the proud position that she holds in the world. It is, therefore, urgently necessary that some remedy should be found, and in disease it is useless to treat symptoms only, we must attack the cause.

Without going into more detail, let us say that the great principles underlying health are: Fresh Air—not produced by artificial fans, but by the windy fan of Nature; Adequate, Nourishing Food, not artefacts; Daily Outdoor Exercise, with plenty of pleasurable games, followed by regular, natural sleep. To obtain these necessary conditions a system of garden cities appears to offer the best solution; many a war allotment garden has been of considerable benefit to the health of its owner. We think that this system of allotments should be largely extended; it provides a welcome and healthy respite for old and young; . . . and certainly it helps to cut down the weekly household expenditure. Healthy exercise produces natural sleep, and this latter is absolutely necessary for health. All vegetables grown on these allotments could be eaten fresh and uncontaminated by any other hands than those of the grower. Sleeping out of doors during summer should, and could, then be encouraged.

### Results to be Obtained.

How readily would these changes bring back a healthy colour to many a pallid face? Again, the further afield that factories are built from cities then the healthier and happier will be the workers in that factory, and, therefore, the better work will they do—and this is a pressing need of the country to-day.

They will not need to spend much valuable time travelling in close and unhealthy vehicles, and will be able to enjoy the great benefits of the pure open air immediately on leaving their work.

We have seen much experimental evidence regarding efficiency-producing factors within the factories, the methods of increasing output and yet improving the health of the labourer. There is invaluable work to be done thus internally, and already we see the good effects produced, but such hard-earned progress would be as little compared with the results we might expect from a working people who nightly enjoyed the fresh air of the open country-side.

